# The role of parasitism in the energy management of a free-ranging bird

**DOI:** 10.1242/jeb.190066

**Published:** 2018-12-12

**Authors:** Olivia Hicks, Sarah J. Burthe, Francis Daunt, Mark Newell, Olivier Chastel, Charline Parenteau, Jonathan A. Green

**Affiliations:** 1 School of Environmental Sciences, University of Liverpool, Liverpool L69 3GP, UK; 2 Centre for Ecology & Hydrology, Bush Estate, Penicuik, Midlothian, EH26 0QB, UK; 3 Centre d’Etudes Biologiques de Chizé, UMR 7372 – CNRS & Université de La Rochelle, FR-79360 Villiers en Bois, France

**Keywords:** Accelerometry, Daily energy expenditure, Immune costs, Resting metabolic rate, Thyroid hormones, Trade-off

## Abstract

Parasites often prompt sub-lethal costs to their hosts by eliciting immune responses. These costs can be hard to quantify but are crucial to our understanding of the host's ecology. Energy is a fundamental currency to quantify these costs, as energetic trade-offs often exist between key fitness-related processes. Daily energy expenditure (DEE) comprises of resting metabolic rate (RMR) and energy available for activity, which are linked via the energy management strategy of an organism. Parasitism may play a role in the balance between self-maintenance and activity, as immune costs can be expressed in elevated RMR. Therefore, understanding energy use in the presence of parasitism enables mechanistic elucidation of potential parasite costs. Using a gradient of natural parasite load and proxies for RMR and DEE in a wild population of breeding European shags (*Phalacrocorax aristotelis*), we tested the effect of parasitism on maintenance costs as well as the relationship between proxies for RMR and DEE. We found a positive relationship between parasite load and our RMR proxy in females but not males, and no relationship between proxies for RMR and DEE. This provides evidence for increased maintenance costs in individuals with higher parasite loads and suggests the use of an allocation energy management strategy, whereby an increase to RMR creates restrictions on energy allocation to other activities. This is likely to have fitness consequences as energy allocated to immunity is traded off against reproduction. Our findings demonstrate that understanding energy management strategies alongside fitness drivers is central to understanding the mechanisms by which these drivers influence individual fitness.

## Abstract (precis)

**Summary:** Demonstration of the sub-lethal costs of endo-parasitism for a wild population of seabirds via increased maintenance metabolism.

## INTRODUCTION

Parasites cause major fitness consequences to a huge array of taxa (Booth et al., 1993; Gooderham and Schulte-Hostedde, 2011; Reed et al., 2008). Often, parasites prompt sub-lethal effects to the host by eliciting immune or stress responses (Lettini and Sukhdeo, 2010; Martin et al., 2003; Sheldon and Verhulst, 1996; Smyth and Drea, 2016). However, the costs of these sub-lethal effects can be hard to quantify and consequently their impact on individuals and populations is often neglected in ecological research (Binning et al., 2017). Additionally, the mechanisms underlying the fitness effects of parasitism and individual variation in these effects are not well understood. Ignoring these effects of parasites reduces our understanding of their host's ecology, as we know that parasites can drive a large array of fitness-related traits (Boulinier et al., 2016; Hamilton and Zuk, 1982; Norris and Evans, 2000; Reed et al., 2008; Sheldon and Verhulst, 1996).

**Fig. 1.**
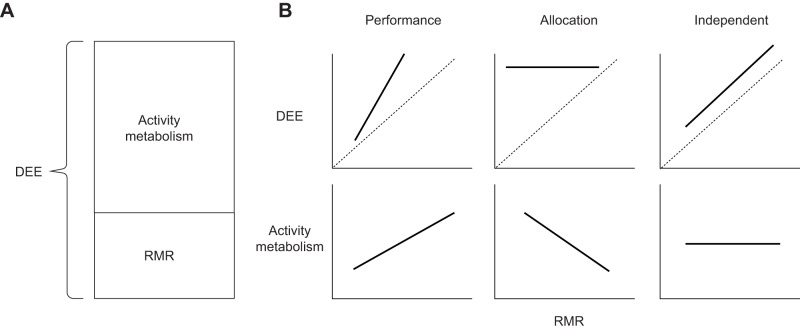
**Graphical representation of energy budgets and possible energy management strategies.** (A) Daily energy expenditure (DEE) is the sum of resting metabolic rate (RMR) and activity metabolism. (B) Three theorized energy management strategies in graphical form adapted from Mathot and Dingemanse (2015) and Careau and Garland (2012) showing the relationship between RMR and DEE and activity metabolism under the three scenarios. The dashed 1-to-1 line is presented to aid interpretation of the slope of the lines. It represents a theoretical situation under which animals are completely inactive and thus DEE is equal to RMR.

Energy can be a limited resource, and individuals are required to allocate energy to the demands of competing life-history traits to maximize fitness (Stearns, 1992). Therefore, trade-offs may exist in energetic terms between key fitness-related behaviours and processes, and so energy is a central mechanism by which fitness responses to parasitism are manifested. A fundamental potential trade-off exists between basal or resting metabolic rate (RMR) and the amount of energy apportioned to activity, because ceilings can exist on the sum of these two components: the daily rate of energy expenditure (DEE) (Careau and Garland, 2012; Elliott et al., 2014; Mathot and Dingemanse, 2015; Welcker et al., 2010). For endotherms, RMR is usually defined as the minimum energetic cost of living during thermo-neutral rest in a free-ranging animal (Mathot and Dingemanse, 2015; Welcker et al., 2015) and therefore largely represents the cost of self-maintenance, including immune activity (Burton et al., 2011). The remaining energy that is available to allocate to activity is referred to as either metabolic scope or activity metabolism (Careau and Garland, 2012; Mathot and Dingemanse, 2015). DEE, RMR and activity metabolism are linked in different ways depending on which energy management strategy the animal is operating under; namely, the performance, independent or allocation strategies (see Fig. 1 and Appendix for details). A recent review of energy management in birds and mammals suggests that no single energy management strategy operates across all species (Portugal et al., 2016).

Parasitism may play a central role in the relationship between self-maintenance and activity because a primary predicted cost of parasitism is increasing RMR, through immune-associated costs (Svensson et al., 1998). Hosts should invest in immunity to reduce the impacts of parasitism, particularly in long-lived species, in which, in contrast to short-lived species, survival contributes more to fitness, and individuals should be selected to allocate more resources to a rapid, strong immune response to combat cumulative damage from parasites and to protect future reproductive success (Lochmiller and Deerenberg, 2000; Moe et al., 2007). Under the allocation model of energy management, this investment would create a trade-off between immune activity and other essential fitness-related organismal functions (Råberg et al., 2000; Sheldon and Verhulst, 1996). Under the performance model, there would be no trade-off with increased RMR, but instead activity metabolism would also increase with a resultant relatively large increase in DEE. Likewise, under the independent model, despite no relationship between RMR and activity metabolism, an increase in RMR results in an equivalent increase in DEE, since this is the sum of activity metabolism and RMR (Mathot and Dingemanse, 2015; Welcker et al., 2015). The lack of a trade-off would itself likely have implications for fitness, and animals might ultimately reach maximal or optimal limits of DEE (Careau and Garland, 2012; Mathot and Dingemanse, 2015).

Understanding the impacts of natural parasite burdens on individual metabolic rate is crucial, with experimental studies suggesting that endo-parasites and elevated immune function incur significant costs to hosts (Albon et al., 2002; Newborn and Foster, 2002; Sheldon and Verhulst, 1996). However, there has been little work on the role of parasitism in mediating the energy management of individuals, despite this being fundamental to the understanding of the fitness effects of parasitism. This is in large part due to the difficulties in measuring natural parasite loads, particularly endo-parasites, in wild populations. Energetic rates are also hard to measure in free-ranging animals and so various proxies are used to evaluate energetic costs (Butler et al., 2004; Chastel et al., 2003; Halsey et al., 2011).

Here, we measure proxies of both DEE and RMR in individuals with known natural endo-parasite loads in a free-living population of breeding European shags [*Phalacrocorax aristotelis* (Linnaeus 1761)] to understand the effect of endo-parasites on maintenance costs as well as the relationship between maintenance and total energy expenditure under high energetic demands during the breeding period. Specifically, we investigate two questions: (1) Does parasite load relate to RMR? We expect that individuals with higher parasite loads will have higher immune costs and that this will be reflected in elevated RMR. (2) Does DEE relate to RMR and what can this tell us about the allocation between energy to reproduction and self-maintenance of this species? We predict that individuals will be energetically constrained by rapidly growing chicks during the chick-rearing period under the allocation model and therefore any increase in RMR will not result in an increase in DEE.

## MATERIALS AND METHODS

### Study site and species

The study was carried out at the Isle of May National Nature Reserve, south-east Scotland (56°11′N, 2°33′W) during the breeding seasons of 2015–2017. All individuals were part of a long-term population study and are individually marked with a unique metal ring to allow accurate aging of individuals that were first ringed as chicks and a coded plastic ring to facilitate identification from a distance.

Previous sampling of this population has shown a high prevalence of the nematode parasite *Contracaecum rudolphii* Hartwich 1964 (Burthe et al., 2013; Granroth-Wilding et al., 2014; Reed et al., 2008) in European shags, although parasite loads vary markedly between individuals (Burthe et al., 2013; Granroth-Wilding et al., 2014; Reed et al., 2012). Shags become infected with third-stage larvae via their fish diet. Larval worms moult to become sexually mature adults, which attach to the lining of the proventriculus and lower oesophagus in the final seabird host (Abollo et al., 2001; Burthe et al., 2013).

### Estimating resting metabolic rate

Measuring RMR in the field is difficult (Wilson et al., 2006). Our previous work using calibrated estimates of RMR from accelerometry (Hicks et al., 2017) suggested that RMR of European shags is linked to parasite burden (Hicks et al., 2018). However, despite evidence that movement costs from accelerometry can indeed be used to evaluate costs during relative inactivity (Green et al., 2009), we wanted to use a more sensitive proxy for RMR to investigate this effect further. Thyroid hormone plasma concentrations are increasingly being used in this context in free-ranging animals. The role of thyroid hormones in energy metabolism in birds in particular is now well established (Chastel et al., 2003; Elliott et al., 2013; Welcker et al., 2013). Triiodothyronine hormone (T3; 3,3′,5-triiodo-L-thyronine) especially is considered one of the major controllers for the regulation of tissue oxygen consumption and metabolic activity in endotherms (McNab, 1997). Studies carried out both in the laboratory and the field show close relationships for a range of species and life-history strategies (e.g. Chastel et al., 2003; Duriez et al., 2004; Elliott et al., 2013; Vézina et al., 2009; Welcker et al., 2013; Zheng et al., 2014). While a direct calibration of T3 hormone and RMR has yet to be conducted for our study species, we are unaware of any calibration studies during breeding which have failed to establish such a relationship. Thus, we feel confident that T3 hormone can be used as a proxy for RMR at the individual level, without the confounding effect of stress associated with respirometry (Welcker et al., 2015).

Adult European shags were captured on the nest during chick rearing (when the chicks were between 5 and 36 days old) using a crook on the end of a long pole. At capture, a 1 ml blood sample was collected from the brachial vein using a heparinized syringe and a 25-gauge needle. To avoid an effect of handling stress on hormone concentrations, sampling took place within 3 min of capture. Blood samples were taken between 03:30 and 07:30 h, before shags left for their first foraging trip of the day, and therefore birds were assumed to be post-absorptive, which was later confirmed by endoscopy. Throughout the fieldwork period, local air temperature was comfortably above the estimated lower critical temperature of 6°C for European shags (Enstipp et al., 2005); thus, birds were in thermoneutrality at all times. Blood samples were stored on ice in the field. Whole blood was centrifuged and plasma and red blood cells were kept frozen at −20°C until subsequent analyses.

Total T3 hormone assays were performed at the Centre d'Etudes Biologiques de Chizé (CEBC), France. T3 analyses were performed using a single radioimmunoassay (RIA) and total thyroid hormone levels were assessed in duplicate without extraction as in Chastel et al. (2003) and Welcker et al. (2013). A total of 25 µl of plasma was incubated with 10,000 cpm of 125I-hormone (Perkin Elmer) and polyclonal rabbit antiserum (Sigma-Aldrich). The bound fraction was separated from the free fraction by addition of a sheep anti-rabbit antibody. After overnight incubation and centrifugation, the bound fraction activity was then counted on a Wizard 2 gamma counter (Perkin Elmer). For quality control, reference materials were used and were within the acceptable range determined by the laboratory. Furthermore, every plasma was run in duplicate, and samples with a coefficient of variance (CV) higher than 12% were re-assayed. Inter- and intra-assay variations were, respectively, 14.34 and 7.75%. Cross-reactions of T3 antiserum were as follows: triiodo-D-thyroacetic acid 6%, L-thyroxine 0.2%, diiodo-L-thyrosine <0.01%, monoiodo-L-thyrosine <0.01%. Cross-reactions of T4 antiserum were as follows: triiodothyronine 4%, diiodo-L-thyrosine <0.01%, monoiodo-L-thyrosine <0.01%.

### Quantifying parasite load

Worms were counted visually using the endoscope video screen (for detailed endoscopy methods, see Burthe et al., 2013). Burdens higher than 40 worms were hard to quantify in the field accurately and these were counted retrospectively via endoscopy video footage. Quantification of parasite burdens was found to be repeatable within an individual across a season (Burthe et al., 2013). All endoscopy and blood sampling was performed by trained personnel (S.J.B.) holding a personal licence, and under a project licence issued by the UK Home Office.

### Estimating daily energy expenditure

Tri-axial accelerometers (little Leonardo D3GT, AXY3 and Gulf Coast Data Concepts X8) were used to estimate DEE in a subset of the birds used for the hormone assay. Accelerometers were set to record at 25 or 50 Hz and attached on the midline of the mid back of individuals (as close to the centre of gravity as possible) using Tesa tape. All birds were successfully recaptured and accelerometers were retrieved after 3 days of deployment. Energy expenditure was estimated for diving, flying and resting [the main activities of European shags (Sakamoto et al., 2009)] using behaviour-specific calibrations derived from respirometry calibrations with heart rate and accelerometry (see Hicks et al., 2017 for behaviour-specific calibrations and detailed methods). These data were then used to estimate total DEE, the sum of the energetic costs of all behavioural bouts within 24-h periods of activity in terms of the rate of oxygen consumption (l day^−1^). This included both activity and RMR and any costs of thermoregulation in water (see Hicks et al., 2018 for full details). Adult RMR and DEE have been found to vary with mass, reproductive stage and age in many seabirds (Elliott et al., 2014; Green et al., 2013; Grémillet, 1997; Weimerskirch et al., 1995). Thus, adult mass and age were recorded at endoscopy and chick age at time of sampling was later back-calculated from wing length at ringing (Granroth-Wilding et al., 2014).

### Statistical analysis

All models were fitted separately for males and females due to non-independence of nest pairs. To answer the first question of whether parasite load is related to elevated RMR, we modelled T3 concentration using linear mixed effects models (LMMs). Parasite load, brood size (number of chicks), brood age (age of the oldest chick in the brood at the time of sampling), year, and adult age and body mass were explanatory variables and we controlled for variation between birds and repeated sampling by including individual as a random factor. We fitted models containing all combinations of the fixed effects, and interactions between parasite load and brood age and parasite load and brood size (see Table 1 for explanations of model terms).

**Table 1.**
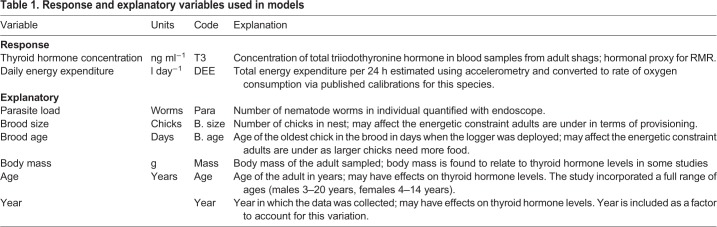
**Response and explanatory variables used in models**

Secondly, to understand how variation in RMR is related to DEE, we modelled estimated DEE as a function of T3 concentration (a proxy for RMR) using LMMs. Adult age, adult body mass, brood age (at the time of sampling), brood size and year were also included as explanatory variables (Table 1). We controlled for variation between birds and repeated sampling by including individual as a random factor.

All models were fitted using the lme4 package in R (Bates et al., 2014; http://www.R-project.org/) and model selection for both sets of models was based on Akaike's information criterion (AIC) (Burnham and Anderson, 2001).

## RESULTS

### Relationship between parasite load and RMR

**Fig. 2.**
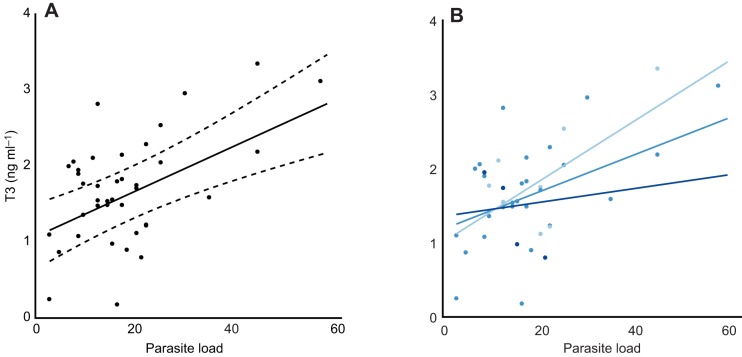
**Relationship between parasite load and plasma total 3,3′-triiodo-L-thyronine (T3) concentration (a proxy for RMR) in female European shags.** Data points represent individual T3 concentrations, the predicted lines from the best-supported model (solid lines) and their 95% confidence intervals (dashed). (A) The relationship between parasite load and T3 concentration when year and brood age are accounted. (B) The interaction between parasite load and brood age in relation to T3 concentration, the explanatory terms in the best-supported model. Solid lines represent predicted lines from the best-supported model under different brood age scenarios −1 s.d. of the mean brood age (light blue), mean brood age (mid-blue) and +1 s.d. of the mean (dark blue).

**Fig. 3.**
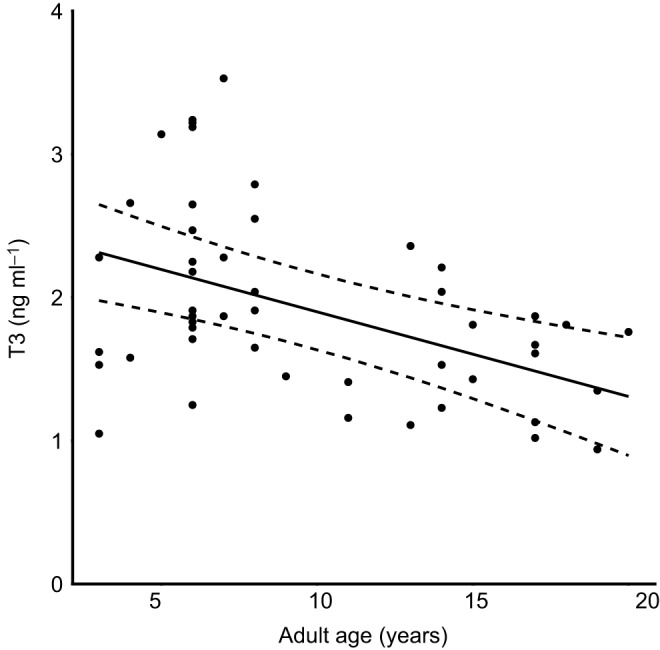
**Significant relationship between adult age and plasma total T3 concentration (a proxy for RMR) in male European shags.** Data points represent individual T3 concentrations, solid lines the predicted lines from the best-supported model and dashed lines their 95% confidence intervals.

A total of 87 (48 males and 39 females; 2015: 8, 2016: 42, 2017: 37) individuals were blood sampled and endoscoped over 3 years (of which 27 were sampled in more than one year). Whilst the sample size in 2015 is clearly smaller than the other years, removal of these data had no impact on the conclusions of the analyses and were therefore included. The best-supported model for females showed a positive relationship between parasite load and T3 concentration, an effect of year, and an interaction between brood age and parasite load in that there was a more positive relationship between parasite load and T3 when broods were younger (see Fig. 2 and Table 2). When year and brood age are accounted for, T3 concentration increased by 150% across the range of natural parasite load. The best-supported model for males showed no effect of parasite load on T3 concentration. There was a negative effect of age as well as an effect of year on T3 concentration in males (see Fig. 3 and Table 2).

**Table 2.**
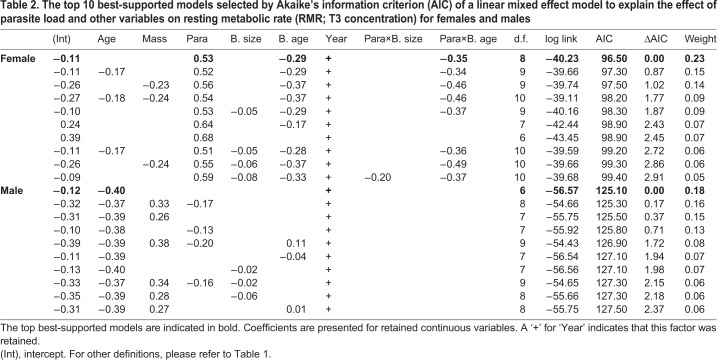
**The top 10 best-supported models selected by Akaike's information criterion (AIC) of a linear mixed effect model to explain the effect of parasite load and other variables on resting metabolic rate (RMR; T3 concentration) for females and males**

### Relationship between proxies for RMR and DEE

**Fig. 4.**
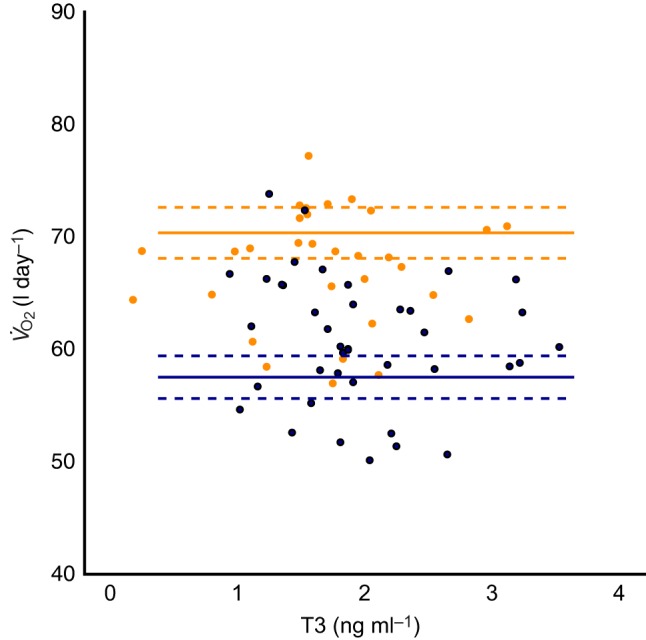
**Relationship between plasma total T3 concentration (a proxy for RMR) and estimated DEE in terms of rate of oxygen consumption in European shags.** Dots represent individual T3 concentrations. The predicted lines from the best-supported model (solid lines) and their 95% confidence intervals (dashed) are presented for males (blue) and females (orange). *V̇*_O_2__, rate of oxygen consumption.

DEE was estimated in a subset of the birds (71 individuals; 2015: 3, 2016: 40, 2017: 28) to gain insight into the relationship between RMR and DEE. The best-supported model for females was the null model with no relationship found between T3 and estimated DEE (see Fig. 4 and Table 3). For males, the best-supported model indicated a negative effect of body mass on estimated DEE and no effect of T3 (see Fig. 4 and Table 3). However, the null model is also equally well supported (within Δ2 AIC of the top model); thus, there is very little support for an effect of mass.

**Table 3.**
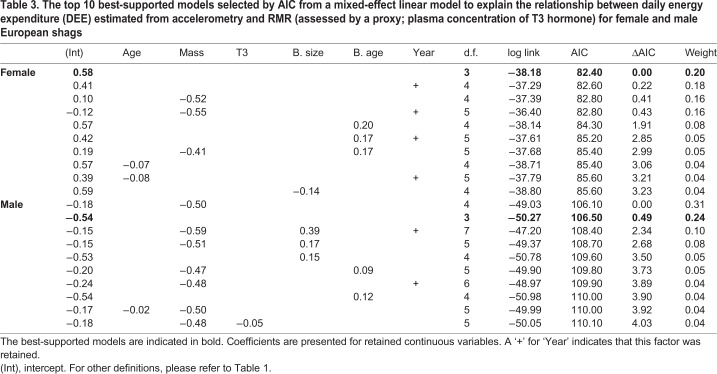
**The top 10 best-supported models selected by AIC from a mixed-effect linear model to explain the relationship between daily energy expenditure (DEE) estimated from accelerometry and RMR (assessed by a proxy; plasma concentration of T3 hormone) for female and male European shags**

## DISCUSSION

Measuring both natural parasite burdens and metabolic rates in free-ranging animals is challenging. In particular, estimating metabolic rate requires the use of proxies, the potential limitations of which have been extensively documented (e.g. Butler et al., 2004; Halsey et al., 2011). Our study builds on previous work (Hicks et al., 2018) to further explore links between RMR and parasite loads in European shags and how these might link to DEE. Despite the potential limitations, we found a positive relationship between natural parasite load and our hormonal proxy for RMR across multiple years in female shags, strongly suggesting increased maintenance costs in individuals with higher parasite loads. Host maintenance costs are likely to be increased in response to parasitism due to increased cell repair and cost of dealing with the parasite infection as well as the immune response itself (Lochmiller and Deerenberg, 2000). The limitations of the approaches used would have made it difficult to distinguish between the independent and performance strategies, since our proxies for RMR and DEE use different units of measurement. However, the lack of relationship between these two proxies provides strong evidence that female European shags operate under an allocation energy management strategy. Therefore, a heightened immune response to parasitism creates restrictions on the allocation of energy to other activities and is likely associated with subsequent fitness consequences.

We found no relationship between our proxy for RMR and parasitism in males despite the strong relationship found in females. Males are hypothesized to experience the immunosuppressive qualities of testosterone (Grossman, 1985); thus, the lack of relationship may be due to suppression of their parasite-induced immune response. Additionally, trade-offs between reproduction and self-maintenance are especially strong in female shags (Hicks et al., 2018; Reed et al., 2008), who have higher estimated DEE than males on average (see Fig. 3 and Hicks et al., 2018) and can be more adversely affected by environmental conditions than males (Lewis et al., 2015). Females across many taxa often invest more in reproduction than males (Clutton-Brock, 1991), meaning that females may be closer to their energy expenditure limits than males. Thus, the greater energetic constraint of females to extrinsic and intrinsic conditions could explain these sex differences in response to parasitism (Hicks et al., 2018; Lewis et al., 2015).

This study is the first to use T3 concentration as a proxy for RMR in the context of parasitism effects. This enables us to gain greater insight into the energy use of free-ranging animals and complements our previous findings and other approaches. Hicks et al. (2018) show that, for female European shags, flight costs are positively associated with parasite burden but that the proportion of time spent in flight per day reduced with higher parasite loads. However, this decrease in flight duration was more than expected based on the increased cost of flight alone. The results from this study suggest that the reduction in flight duration observed in heavily parasitized birds may be due to compensation for increased maintenance costs as well as the increased cost of flight (Hicks et al., 2018). For European shags, it is not possible to quantify the extent of this compensation as we have yet to calibrate T3 concentration against RMR using respirometry. However, T3 plasma concentration is increasingly being used as a proxy for RMR due to strong correlations found in multiple species and the reduction in the confounding effect of stress associated with respirometry (Blackmer et al., 2005; Bouwhuis et al., 2011; Chastel et al., 2003; Elliott et al., 2013; Moe et al., 2007; Welcker et al., 2013, 2015). In an experimental study, a 50% increase in free T3 corresponded to a 50% increase in RMR in kittiwakes (Welcker et al., 2015). In this study, we found a 150% change in T3 concentration across the natural range of parasite load, meaning that the corresponding change in RMR to parasitism could be even greater than in previous experimental work.

It is likely that energy management is not fixed across time but varies according to particular conditions, as seen in Hicks et al., (2018) where estimated DEE increased with brood age but not in relation to parasite load. An adult's cumulative energetic investment in its brood increases with brood age (Drent and Daan, 1980). Thus, the trade-off between allocation to reproduction and self-maintenance may also shift with brood age. In a young brood, when cumulative investment is lower than in an older brood, energy allocation to immune response may be beneficial to ensure that the next reproductive event is reached (Ilmonen et al., 2000), but may reduce parental effort (Råberg et al., 2000) and negatively affect offspring success. However, when cumulative investment in a brood is higher, investment to current reproduction may be prioritised. Consistent with this concept, we found that the positive relationship between RMR and parasite load is greater when the adult's chicks are younger, suggesting that the shags are investing more in parasite resistance at this time.

At the scale of a lifetime, life-history theory predicts that trade-offs between reproduction and self-maintenance change with age. As the probability of future reproduction declines with age, it is predicted that resources will be increasingly allocated to current reproduction rather than maintenance (Herborn et al., 2016; van Noordwijk and de Jong, 1986). There is good evidence for this in short-lived species such as great tits and zebra finch (Bouwhuis et al., 2011; Moe et al., 2009); however, in longer-lived birds, evidence is equivocal (e.g. Blackmer et al., 2005; Elliott et al., 2015; Moe et al., 2007). In this study, we found a negative relationship between age and RMR in males, suggesting that older individuals invest less energy in maintenance, as in thick-billed murres (Elliott et al., 2015). However, we found no effect of age on RMR in females. Sex-specific senescence occurs in a number of wild vertebrate populations, possibly due to different trade-offs existing with age in males and females (Clay et al., 2018; Froy et al., 2013, 2017; Murgatroyd et al., 2018).

We found no evidence for a relationship between proxies for RMR and DEE, indicating that DEE does not vary with maintenance costs. Despite parasite-induced behavioural costs, European shag estimated DEE does not correlate with parasite load (Hicks et al., 2018), suggesting that there is a level of energy expenditure for this population that they are unwilling or unable to exceed. Drent and Daan (1980) first introduced the concept of an ‘optimal working capacity’ as a rate beyond which parents would increasingly suffer from any kind of risks or dangers with the consequence that their lifetime reproductive success would be reduced (Green et al., 2009). Evidence for such an optimum, occasionally described as an ‘energetic ceiling’, have been described previously (e.g. Welcker et al., 2010) and provide evidence for the existence of allocation management strategies. Our results also provide evidence for both an optimum to energy expenditure and the allocation management strategy in European shags (Drent and Daan, 1980; Mathot and Dingemanse, 2015). As such, an increase in RMR will cause a reduction in activity metabolism, which may have consequences for fitness-related behaviours such as chick provisioning or foraging. Our findings are in agreement with studies providing evidence for the allocation management strategy in three seabird species (Blackmer et al., 2005; Elliott et al., 2014; Welcker et al., 2015) but are contrary to other studies (including those of other seabird species) providing evidence for both performance and independent strategies (Chastel et al., 2003; Portugal et al., 2016).

It is unclear why species vary in their energy management strategy, although, during energetically demanding periods, the capacity to increase expenditure can impose energy limits, in terms of foraging constraints, making an allocation strategy more likely (Elliott et al., 2014). Under limited energy it can be difficult to buffer the additional costs of parasites. Therefore, considering the role of parasitism in mediating energy allocation between reproduction and self-maintenance is crucial in understanding the mechanism of its fitness effects. The allocation strategy creates energetic trade-offs under an overarching constraint yet, before now, we had little knowledge about what drives these trade-offs. Changes in energy allocation can influence reproduction in the short term but in the long term there may be negative consequences of reducing allocation to self-maintenance (Blackmer et al., 2005). Therefore, quantifying energy management strategies alongside potential fitness drivers is crucial to understand the mechanisms by which they act and influence energy allocation to optimize individual success.

## APPENDIX

### Energy management strategies

#### Performance model

Variation in RMR reflects variation in the size of organs that mobilize energy (e.g. digestive organs, muscle). Therefore, individuals with higher RMR are able to maintain higher levels of energy output (i.e. have higher DEE). This strategy predicts higher RMR to be associated with both greater activity metabolism and therefore, when summed, a higher DEE (Careau et al., 2008).

#### Allocation model

Variation in RMR does not reflect variation in the size of organs that mobilize energy; differences in RMR are therefore not associated with differences in DEE. Individuals with higher RMR have less energy available to allocate to energetically costly behaviours and so are predicted to have lower expression of such traits (Mathot and Dingemanse, 2015).

#### Independent model

Regulation of RMR and DEE are independent but an increase in RMR causes an increase in total energy expenditure as activity metabolism is also independent of RMR. Therefore, higher RMR is associated with higher DEE because, although activity metabolism is unaffected, it sums with an increasing RMR to drive an increased DEE.
